# Supplementary material for: 2D material programming for 3D shaping
Source: Nat Commun. 2021 Jan 27;12:603. doi: 10.1038/s41467-021-20934-w (PMC7841157; doi:10.1038/s41467-021-20934-w)
Supplement: Supplementary file 1 — Supplementary Information [file 41467_2021_20934_MOESM1_ESM.pdf]

Supplementary Information

2D material programming for 3D shaping

Amirali Nojoomi[1], Junha Jeon[2] & Kyungsuk Yum[1]*

[1]Department of Materials Science and Engineering, University of Texas at Arlington, Arlington, TX 76019, USA.

[2]Department of Chemistry and Biochemistry, University of Texas at Arlington, Arlington, TX 76019, USA.

*e-mail: kyum@uta.edu

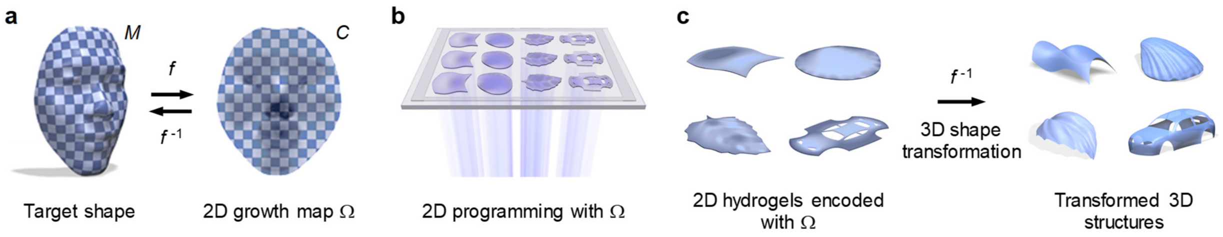

Supplementary Fig. 1. 2D material programming for arbitrary 3D shaping. **a**, Computation of Ω for an arbitrary 3D shape M via conformal mapping f of M onto the plane C. The inverse conformal mapping f^{-1} represents the shape transformation of C to M. **b**, 2D printing of multiple hydrogel sheets encoded with different Ω from a single precursor solution using digital light projection grayscale lithography. **c**, Schematic illustrating 3D shape transformation of 2D hydrogels encoded with Ω to target 3D shapes. The 2D hydrogels reversibly transform to target 3D shapes at the shrunk state upon temperature increase ($T > T_c$), where is T_c is the volume phase transition temperature of pNIPAm hydrogels (~32.5 °C). The experimental 3D structures are shown in Fig. 2.

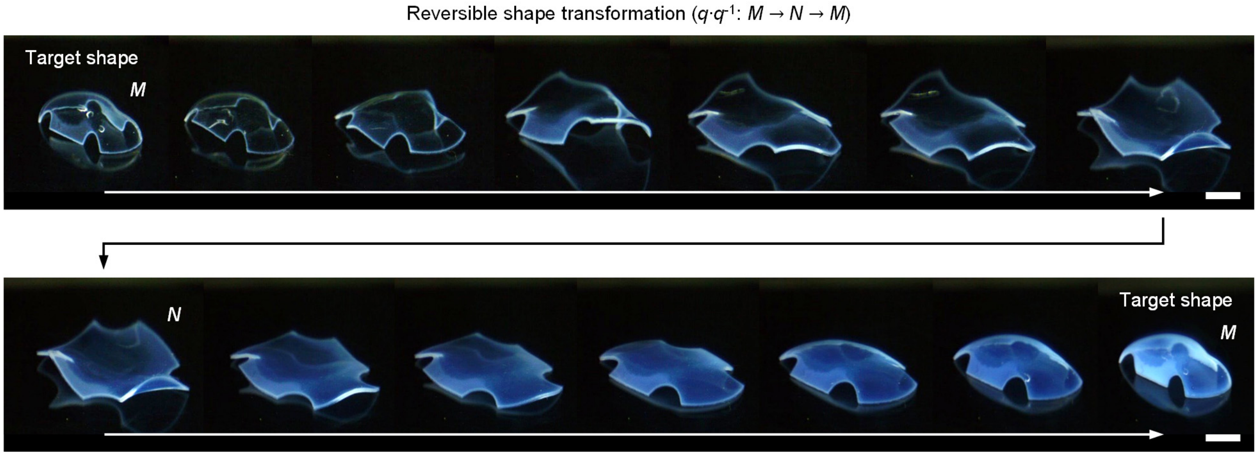

Supplementary Fig. 2. Reversible shape transformation. The structure reversibly transforms between the target shape M at the shrunk state ($T > T_{\mathrm{c}}$) and the shape N at the swelled state ($T < T_{\mathrm{c}}$) upon temperature change. The figure shows the shape transformation from M to N upon temperature decrease ($T < T_{\mathrm{c}}$) and then from N to M upon temperature increase ($T > T_{\mathrm{c}}$) with time (from top left to top right and then from bottom left to bottom right). Scale bar, 4 mm.

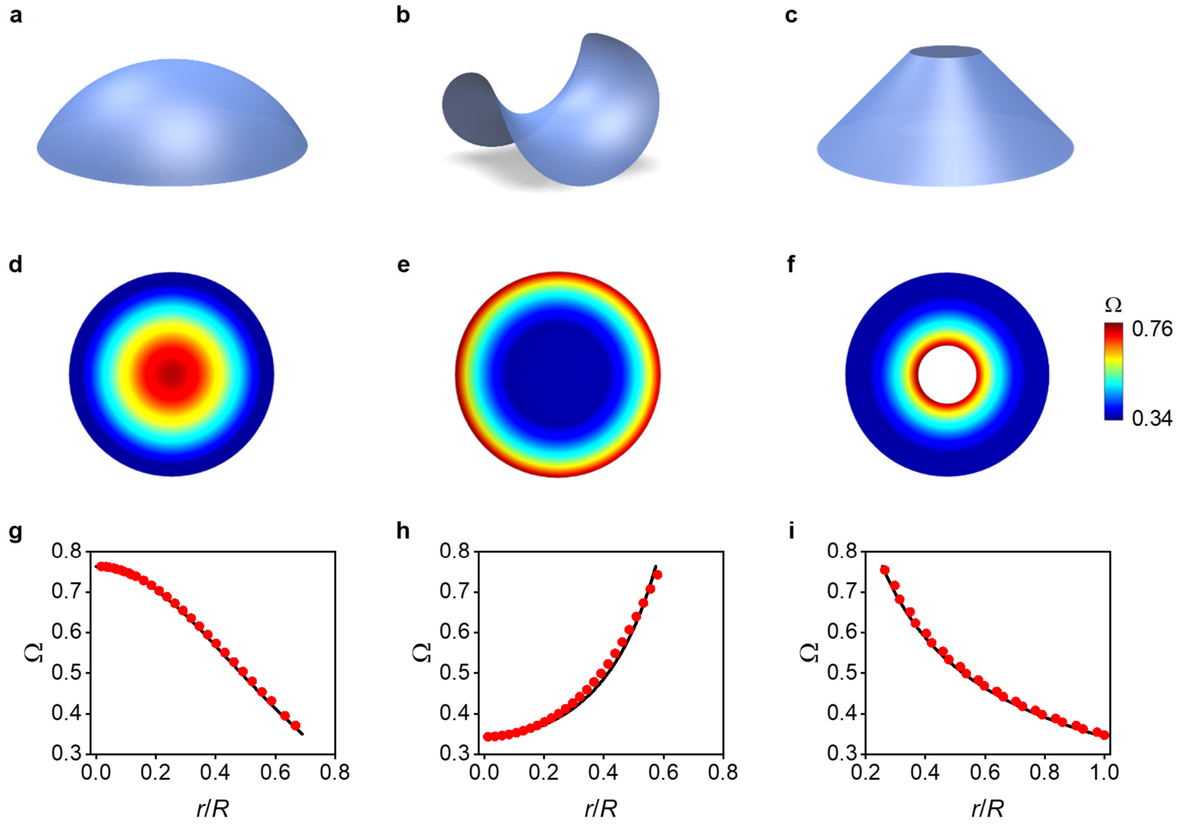

Supplementary Fig. 3. Computation of growth functions Ω for 3D shapes with axisymmetric metrics. **a**–**c**, Target 3D shapes with constant Gaussian curvature K: spherical cap (**a**), saddle (**b**), and cone (**c**) shapes. **d**–**f**, Computed Ω for the special cap (**d**), saddle (**e**), and cone (**f**) shapes in **a**–**c**. **g**–**i**, Ω for the special cap (**g**), saddle (**h**), and cone (**i**) shapes as a function of normalized radial position r/R, where r is a radial position in a polar coordinate system and R is a constant. The filled red circles and the black solid lines indicate computationally obtained and theoretically calculated Ω, respectively.

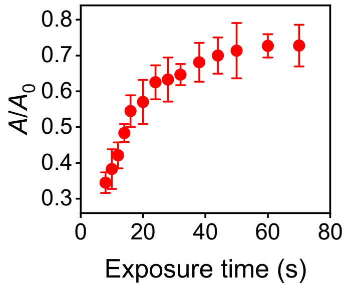

Supplementary Fig. 4. Areal shrinking ratios A/A_0 of dual-crosslinked pNIPAm hydrogels versus light exposure time. A is the area of hydrogels at the shrunk state. A_0 is the area of as-printed hydrogels. The hydrogels were formed with two types of crosslinkers with different lengths: BIS (short-chain crosslinker) and PEGDA (long-chain crosslinker). Error bars represent standard deviation of three independent measurements.

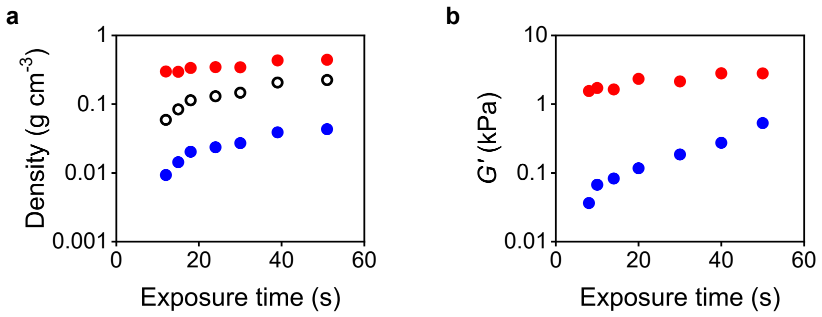

Supplementary Fig. 5. Density and shear storage modulus *G′* of dual-crosslinked pNIPAm hydrogels formed with different light exposure times. **a**, The density of dual-crosslinked pNIPAm hydrogels at the swelled (blue) and shrunk (red) states versus light exposure time. The open black circles indicate the density of as-printed pNIPAm hydrogels prepared with different light exposure times. The density of as-printed pNIPAm hydrogels was calculated using their dry mass and volume. The density of the hydrogels at the swelled and shrunk states was calculated using the density of as-printed pNIPAm hydrogels and the volume swelling and shrinking ratios at 25 °C and 35 °C, respectively. **b**, *G′* of pNIPAm hydrogels at the swelled (blue) and shrunk (red) states versus light exposure time. The figure shows the values of *G′* at an oscillatory frequency of 1 Hz and strain of 1%.

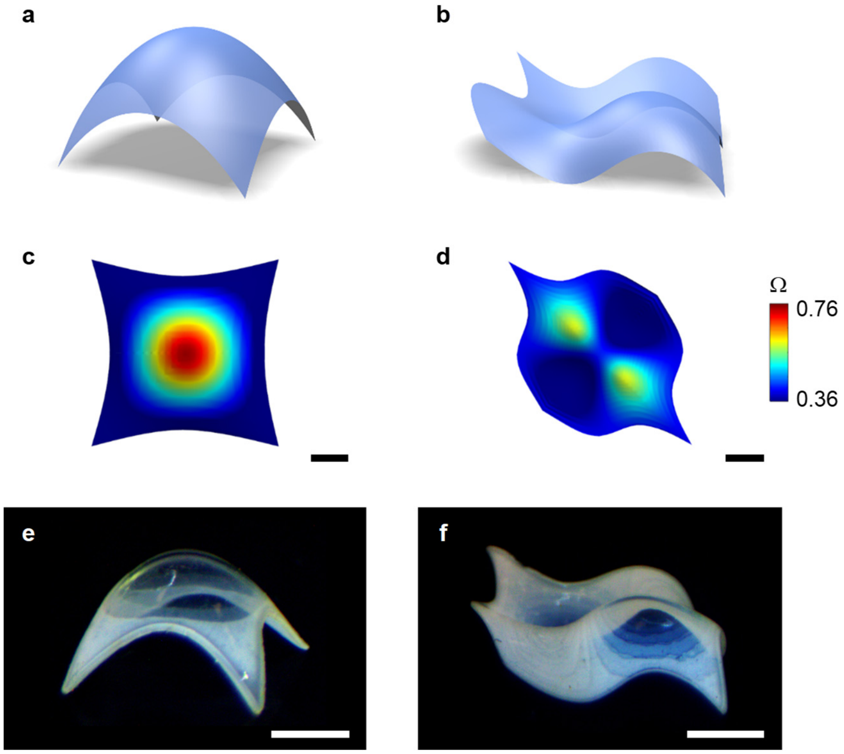

Supplementary Fig. 6. Growth-induced shaping of 3D structures defined by height functions. **a**, **b**, Target 3D shapes defined by height functions $h(x, y) = a(x^m + y^n)$ with a = 0.62, m = 2, and n = 2 (**a**) and a = 1, m = 3, and n = 3 (**b**). **c**, **d**, Ω maps for the target 3D shapes in **a** and **b**. **e**, **f**, Experimentally printed 3D structures using Ω in **c** (**e**) and **d** (**f**). Scale bars, 5 mm.

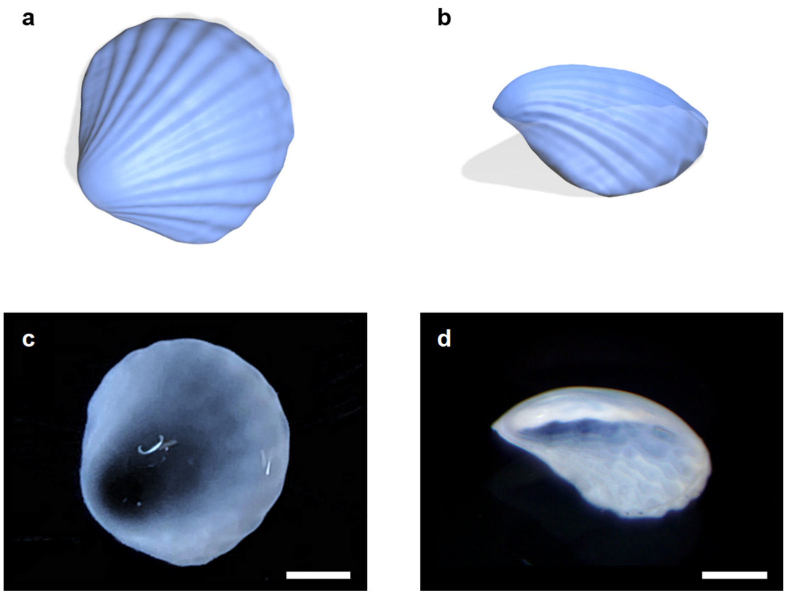

Supplementary Fig. 7. Top and side views of a sea shell structure shown in Fig. 2j. a, **b**, Target 3D shape: top view (**a**) and side view (**b**). **c**, **d**, Experimentally printed 3D structures: top view (**c**) and side view (**d**). Scale bars, 2 mm.

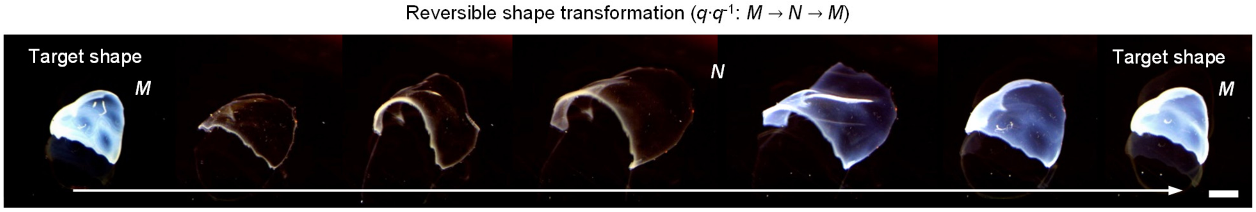

Supplementary Fig. 8. Reversible shape transformation of a leaf structure. The structure reversibly transforms between the target shape M at the shrunk state ($T > T_{\mathrm{c}}$) and the shape N at the swelled state ($T < T_{\mathrm{c}}$) upon temperature change. The figure shows the shape transformation from M to N upon temperature decrease ($T < T_{\mathrm{c}}$) and then from N to M upon temperature increase ($T > T_{\mathrm{c}}$) with time (from left to right). Scale bar, 2 mm.

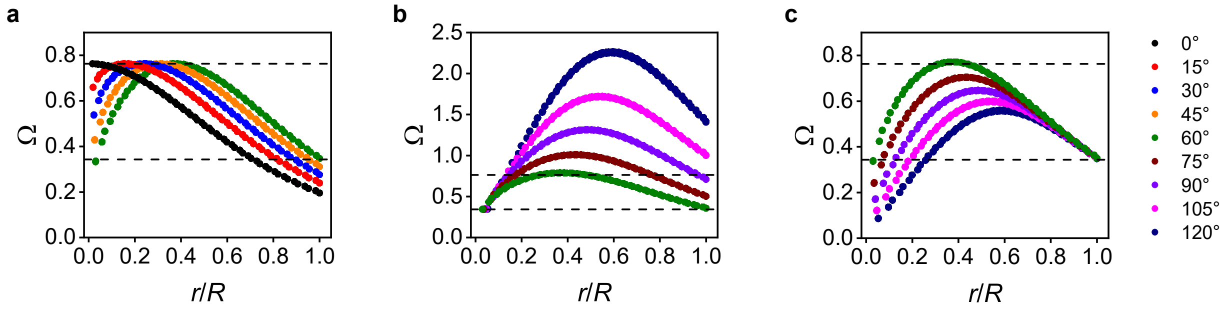

Supplementary Fig. 9. Rescaled Ω for a hemisphere using a cone singularity with different cone angles $\boldsymbol{\theta_c}$. We rescaled Ω shown in Fig. 3c to fit them to the experimentally accessible range of Ω ($0.34 < \Omega < 0.76$) (black dashed lines) with $\Omega_r = 2.22$. We rescaled r/R to have a maximum value of 1, where R is the radius of a 2D hydrogel disk required to form a full hemisphere. We can use the rescaled Ω to experimentally print hemisphere structures (as shown in Fig. 3a and Supplementary Fig. 10). **a**, Ω rescaled to have a maximum value of 0.76 with $0° \leq \theta_c \leq 60°$. The maximum accessible portion of a hemisphere that can be obtained with $0.34 < \Omega < 0.76$ (indicated with black dashed lines), or $\Omega_r = 2.22$, increases with θ_c ($0° \leq \theta_c \leq 60°$). The full range of Ω with $\theta_c = 60°$ is within the experimentally accessible range, thus allowing us to print a full hemisphere as shown in Fig. 3a and Supplementary Fig. 10. We used Ω to print hemisphere structures shown in Fig. 3a and Supplementary Fig. 10. **b**, Ω rescaled to have a minimum value of 0.34 around the cone singularity at $r/R = 0$ with $60° \leq \theta_c \leq 120°$. The maximum accessible portion of a hemisphere that can be obtained with $0.34 < \Omega < 0.76$ (indicated with black dashed lines), or $\Omega_r = 2.22$, decreases with θ_c ($60° \leq \theta_c \leq 120°$). **c**, Ω rescaled to have a minimum value of 0.34 at $r/R = 1$ with $60° \leq \theta_c \leq 120°$. The rescaled Ω induce hemispheres with an opening at the center, where $\Omega < 0.34$. The maximum accessible portion of a hemisphere that can be obtained with $0.34 < \Omega < 0.76$ (indicated with black dashed lines), or $\Omega_r = 2.22$, decreases with θ_c ($60° \leq \theta_c \leq 120°$). In other words, the minimum

accessible size of the opening at the center, in which $\Omega < 0.34$, increases with θ_c ($60° \leq \theta_c \leq 120°$).

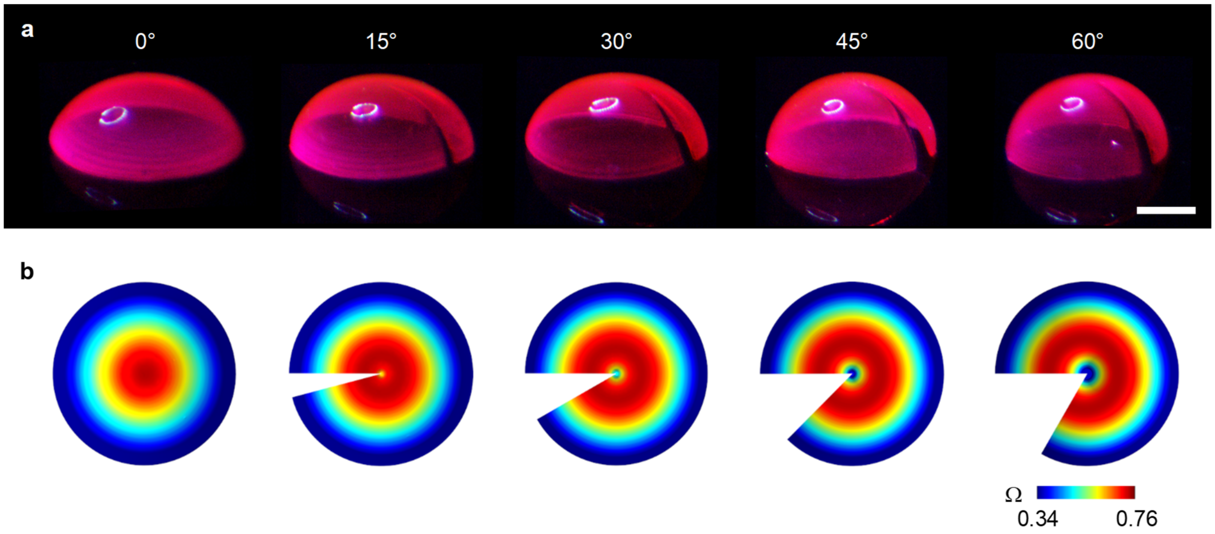

Supplementary Fig. 10. Experimental hemispheres formed a cone singularity with different cone angles $\boldsymbol{\theta_c}$ ($\mathbf{0° \leq \theta_c \leq 60°}$). a, Hemisphere structures obtained by printing 2D hydrogels with a radius of 6 mm using Ω with different θ_c shown in **b**. **b**, Ω maps used to print the structures in **a**. Ω have an axisymmetric functional form at $0° < \theta < 360° - \theta_c$ as shown in Supplementary Fig. 9a, where θ is the angular position in a polar coordinate system.

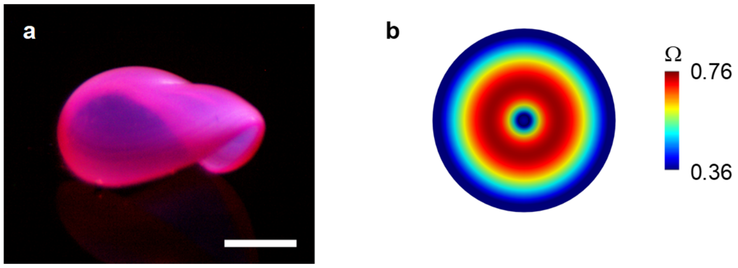

Supplementary Fig. 11. 3D structure (control structure) printed with an axisymmetric Ω without cone singularities obtained from Ω with $\theta_c = 60°$ shown in Fig. 3b, c. **a**, 3D structure printed with axisymmetric Ω in **b**. **b**, Axisymmetric Ω used to print the 3D structure in **a**. We obtained Ω by rotating $\Omega(r/R)$ with $\theta_c = 60°$ (shown in Fig. 3c) from $\theta = 0°$ to $\theta = 360°$, where θ is the angular position in a polar coordinate system. Ω has the form of $\Omega(r/R)$ with $\theta_c = 60°$ in Fig. 3c at $0° < \theta < 360°$. Scale bar, 2 mm.

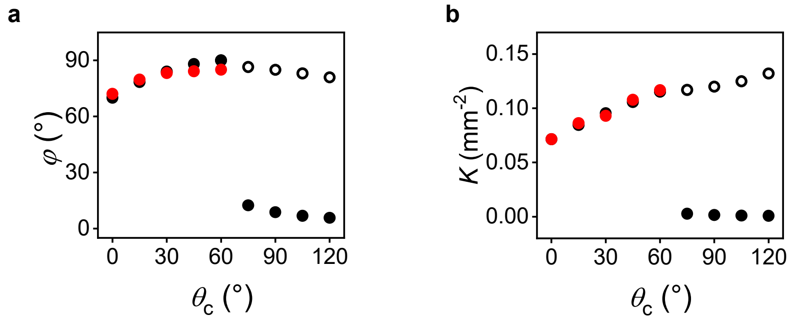

Supplementary Fig. 12. Maximum accessible φ and K of hemispheres that can be obtained with $0.34 < \Omega < 0.76$, or $\Omega_r = 2.22$, using a cone singularity with different cone angles θ_c. φ is the cap angle of a hemisphere, which is the polar angle from the pole of a hemisphere to its base in a spherical coordinate system. **a**, Maximum accessible φ with different θ_c. **b**, Maximum accessible K with different θ_c. The filled red circles and filled black circles at $0° \leq \theta_c \leq 60°$ in **a** and **b** indicate the values of φ and K measured from the experimentally printed structures shown in Supplementary Fig. 10 and those theoretically calculated from Ω shown in Supplementary Fig. 9a, respectively. The filled black circles and open black circles at $60° < \theta_c \leq 120°$ indicate the values theoretically calculated from Ω shown in Supplementary Fig. 9b, c, respectively.

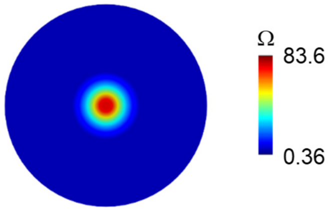

Supplementary Fig. 13. Ω computed for the almost-complete sphere shown in Fig. 3g without cone singularities. Ω has $\Omega_r = 234.3$.

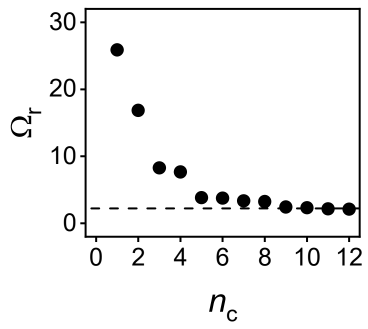

Supplementary Fig. 14. Ω_r required to form the almost-complete sphere in Fig. 3g with different numbers of cone singularities n_c. The dashed line indicates $\Omega_r = 2.22$.

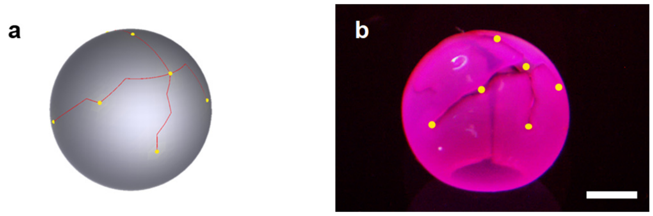

Supplementary Fig. 15. Formation of the almost-complete sphere shown in Fig. 3g with 12 cone singularities. **a**, Target shape with cone singularities (yellow circles) and cutting paths (red lines). **b**, Experimentally printed almost-complete sphere with locations of cone singularities (yellow circles). The locations of cone singularities and cutting paths in the experimental structure in **b** match well with those in the target shape in **a**. Scale bar, 2 mm.

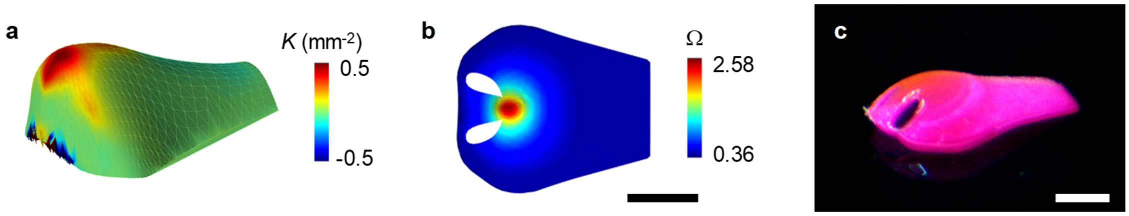

Supplementary Fig. 16. Formation of the nose structure with holes in Fig. 3j using Ω without cone singularities. **a**, Nose model shown in Fig. 3j with K. **b**, Ω computed for the nose model in **a** and Fig. 3j without cone singularities. Ω has $\Omega_r = 7.22$, larger than the accessible Ω_r of our material systems ($\Omega_r < 2.5$). **c**, Nose structure printed using Ω_s obtained by rescaling Ω shown in **b** to the experimentally accessible range of $0.36 < \Omega_s < 0.76$, the same range used to print the nose structure using Ω with a cone singularity in Fig 3k, l: $\Omega_s = (\Omega_{max} - \Omega_{min})(\Omega - \Omega_{min})/(\Omega_r - \Omega_{min}) + \Omega_{min}$, where $\Omega_{max} = 0.76$ and $\Omega_{min} = 0.36$. Scale bars, 5 mm in **b**; 2 mm in **c**.

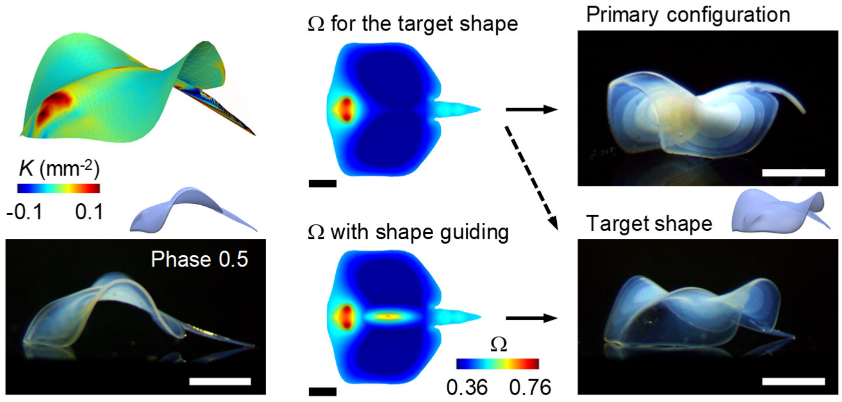

Supplementary Fig. 17. Formation of a stingray structure at phase 0.5 of undulatory swimming motion. The top left panel shows the target shape with K. The bottom left panel shows the side view of an experimentally printed 3D structure (inset: side view of the target shape). The middle panel shows Ω computed for the target shape (top) and Ω with a shape-guiding module with $K > 0$ in the central body (bottom). The right panel shows experimentally printed structures with two types of morphologies: primary configuration induced by Ω (top) and target shape induced by Ω with the shape-guiding module (bottom).

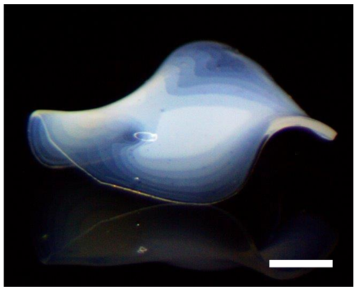

Supplementary Fig. 18. Secondary configuration (configuration 2) of the stingray at phase 0 shown in Fig. 4a. Scale bar, 2 mm.

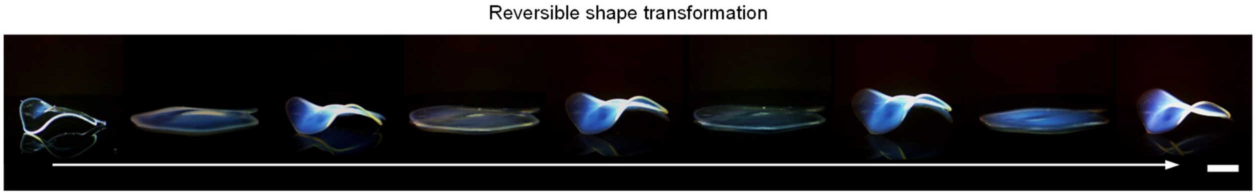

Supplementary Fig. 19. Reversible shape transformation of a stingray structure at phase 0 of undulatory swimming motion. The structure reversibly transforms between the target shape at the shrunk state ($T > T_c$) and the shape at the swelled state ($T < T_c$) upon temperature change. The figure shows multiple cycles of the shape transformation with time (from left to right). The structure was formed using Ω_0 with a shape-guiding module. Scale bar, 5 mm.

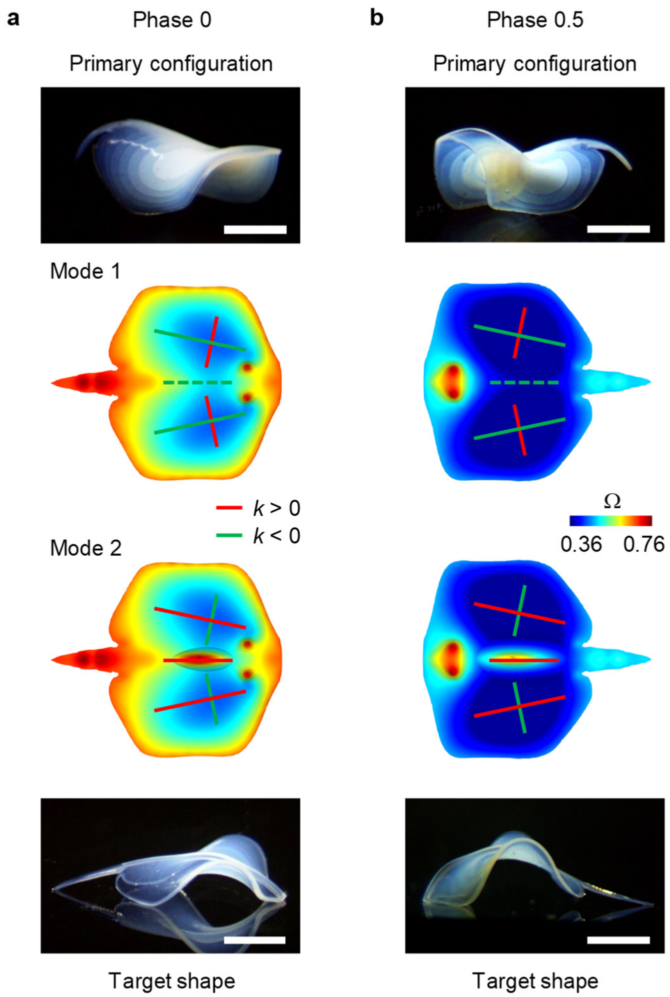

Supplementary Fig. 20. Two major modes of shape morphing of stingray structures. a, Shape morphing modes of a stingray structure at phase 0, which induce the primary configuration (mode 1) and the target shape (mode 2). **b**, Shape morphing modes of a stingray structure at phase 0.5, which induce the primary configuration (mode 1) and the target shape (mode 2). The red and green lines indicate the principal curvatures and directions of the fins, which morph upward ($k > 0$) and downward ($k < 0$), respectively, where k is the curvature. To match the directions of the principal curvatures of stingray structures at phase 0 and phase 0.5, we horizontally flipped the experimentally printed structures and Ω at phase 0 in **a**.

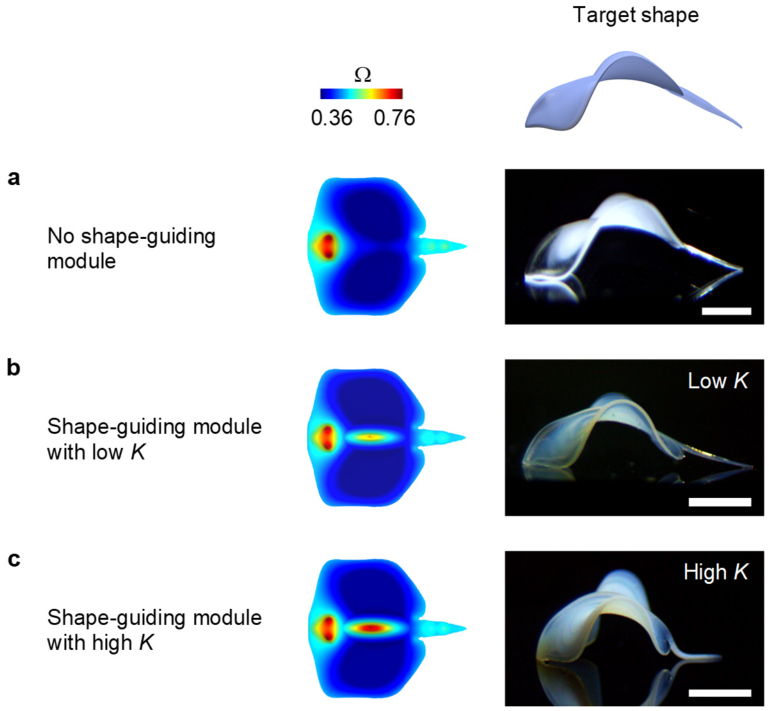

Supplementary Fig. 21. Formation of stingray structures with shape-guiding modules with different *K*. The target shape is the stingray model at phase 0.5 shown in Supplementary Fig. 17. **a**, Stingray structure formed using Ω without shape-guiding modules. **b**, Stingray structure formed using Ω with a shape-guiding module with low K. **c**, Stingray structure formed using Ω with a shape-guiding module with high K. Scale bars, 3 mm.

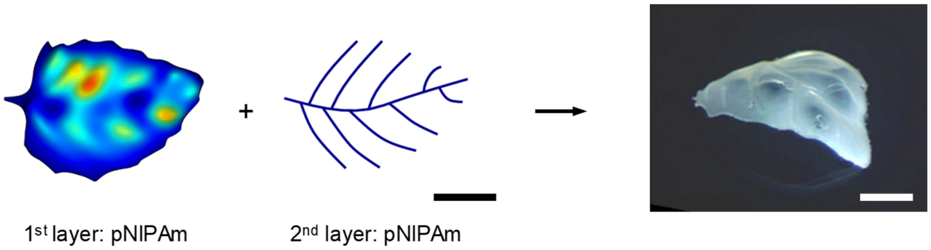

Supplementary Fig. 22. Multilayer 3D structure of a leaf that consists of the lamina (first) and vein (second) layers. We obtained the bilayer structure by sequentially printing the first layer (lamina) and the second layer (veins) with pNIPAm. Scale bars, 5 mm (left); 2 mm (right).

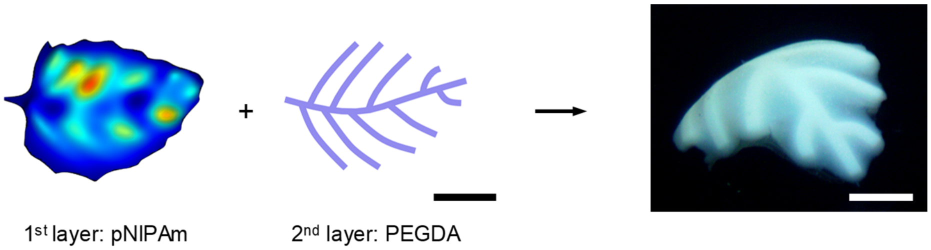

Supplementary Fig. 23. Multilayer 3D structure of a leaf that consists of the lamina (first) and vein (second) layers with two different materials. We obtained the bilayer structure by sequentially printing the first layer (lamina) with pNIPAm and the second layer (veins) with PEGDA. Scale bars: 5 mm.

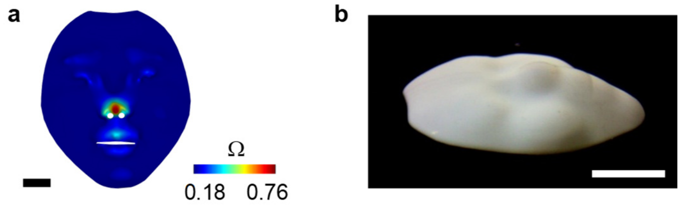

Supplementary Fig. 24. Formation of the real human face in Fig. 4e using Ω without cone singularities. **a**, Ω without cone singularities ($\Omega_r = 4.19$). **b**, Face structure printed using rescaled Ω_s. We obtained Ω_s by rescaling Ω in **a** to the experimentally accessible range of growth of $0.31 < \Omega_s < 0.76$ ($\Omega_r = 2.41$): $\Omega_s = (\Omega_{max} - \Omega_{min})(\Omega - \Omega_{min})/(\Omega_r - \Omega_{min}) + \Omega_{min}$, where $\Omega_{max} = 0.76$ and $\Omega_{min} = 0.31$. The range of growth, $0.31 < \Omega_s < 0.76$, used to print the structure is the same as that used in Fig. 4e. Scale bars, 5 mm.

	Phase 0 (total 6 structures)			Phase 0.5 (total 14 structures)	
	Configuration 1 (Primary)	Configuration 2 (Secondary)	Configuration 3 (Target shape)	Configuration 1 (Primary)	Configuration 2 (Target shape)
Cycle 1	3 (50%)	2 (33%)	1 (17%)	13 (93%)	1 (7%)
Cycle 2	4 (67%)	1 (17%)	1 (17%)	14 (100%)	0 (0%)

Supplementary Table 1. Formation of stingray structures at two phases without shape-guiding modules. We printed 6 and 14 structures at phase 0 and phase 0.5 and observed the formation of 3 and 2 different configurations (isometric embeddings), respectively. We observed the formation of the configurations in the first and second swelling–shrinking cycles as shown in the table.

Supplementary Discussion

Computation of growth functions for target shapes

Given a target 3D shape of a thin sheet with arbitrary complexity, or a surface with Riemannian metric, we determine a 2D growth function Ω required to morph a thin sheet (2D hydrogel) to the target 3D shape (Fig. 1, Supplementary Fig.1). To determine Ω for the target shape, we consider a conformal map $f: M \to C$ from the 3D target shape M with metric g to the 2D plane C with metric $\tilde{g}$. f is conformal if $\langle df(X_1), df(X_2)\rangle = \lambda^2\langle X_1, X_2\rangle$, or $\tilde{g} = \lambda^2 g$, for all tangent vectors X_1 and X_2 at each point on M, where df is the differential of f, $\langle\ ,\ \rangle$ is the inner product of vectors, and λ is a 2D function called the conformal scale factor ($\lambda > 0$)[1]. df expresses how f transforms a tangent vector X on M to a tangent vector $df(X)$ on C. λ quantifies the isotropic change in length of X at each point through f (length growth): $\lambda = |df(X)|/|X|$. The geometric meaning of $\langle df(X_1), df(X_2)\rangle = \lambda^2\langle X_1, X_2\rangle$ is that f only allows uniform (isotropic) scaling of the tangent vectors by λ at each point while preserving the angles between the vectors, which is also the characteristic of isotropic materials (encoded with differential length growth λ).

We therefore postulate that a conformal mapping can describe the in-plane differential growth-induced 3D shaping of isotropic materials: (1) as our hydrogels isotropically swell and shrink, an inverse conformal mapping $f^{-1}: C \to M$ represents an in-plane growth-induced shape transformation of a 2D hydrogel C to a target 3D shape M, or an isometric embedding of M, and (2) λ^{-1} quantifies the length growth (shrinking in this study and thus $0 < \lambda^{-1} < 1$) at each point on the 2D hydrogel required for the shape transformation. We define the area growth function $\Omega = c\lambda^{-2}$ for the target 3D shape, where *c* is a constant. We incorporate the scale factor *c* into Ω to rescale Ω to be within the experimentally accessible range of growth (shrinkage) of our material systems, typically $0.3 < \Omega < 0.8$ at the shrunk state. *c* only rescales the size of the

resulting 3D structure without changing its shape (adopted by Ω), whereas λ^{-2} determines the resulting shape. More rigorously, Ω prescribes the local Gaussian curvature K of the resulting 3D structure, but not necessarily its 3D shape. $K = -\Delta(\ln\Omega)/(2\Omega)$, where Δ is the Laplace operator in the plane[2]. Hence, there are in general multiple isometric embeddings (configurations) of Ω, or the target metric, and therefore multiple configurations that can be induced by Ω (unless there exist constraints). We compute Ω for a target 3D shape by conformally mapping the target 3D shape to the 2D plane using the boundary first flattening (BFF) algorithm[1] with custom MATLAB (MathWorks) code (shown below). The custom code converts $\lambda^{-2}(\xi_1, \xi_2)$ of a target shape obtained from BFF to $\Omega(\xi_1, \xi_2)$ and a STL file, where (ξ_1, ξ_2) are the coordinates on the 2D plane. BFF determines conformal flattening with minimal area distortion by prescribing $\lambda = 1$ along the boundary of a 2D map[1,3]. We also computed Ω with cone singularities using BFF[1].

```
%%%Max of Omega=0.76
%%%Min of Omega=0.36
clear
clc
Scale=dlmread('output file.txt');
O=Scale(:,3);
x=Scale(:,1);
y=Scale(:,2);

%%%%%%%%%%%%%%%%%%%%%%%%%%%%%%%%%%%%%%%%%%%
figure
plot3(x,y,O,'.')
title('O')
MinO=min(min(O))
MaxO=max(max(O))
Orange=MaxO/MinO
Omega=(O/MinO)*0.36; %%% Shift Omega based on the physical limits of materials

%%%%%%%%%%%%%%%%%%%%%%%%%%%%%%%%%%%%%%%%%%%
figure
```

```
plot3(x,y,Omega,'.')
title('Omega')
Omega_Max=max(max(Omega))
Omega_Min=min(min(Omega))

%%%%%%%%%%Trasform Omega to exposure time
expos=(1/(-0.052))*log((Omega-0.805)/(-0.695));%Obtained from the calibration curve
minexposure=min(min(expos))
maxexposure=max(max(expos))
steptime=2;
number_of_slices=maxexposure/steptime

%%%%%%%%%%%%%%%%%%%%%%%%%%%%%%%%%%%%%%%%%%
figure
plot3(x,y,expos,'.')
title('Exposure')
axis([-1,1,-1,1,0,50])
normal_expos=expos/(20*steptime);
solid_expos=surf2solid(x,y,normal_expos,'elevation',0.01);
stlwrite('output file.stl',solid_expos)%Make a STL file
```

Physical realization of conformal mapping $f^{-1}: C \to M$

We physically realize $f^{-1}: C \to M$ with Ω at the shrunk state ($\Omega < 1$) using digital light 4D printing (DL4P)[4] via $f^{-1} = q \cdot p: C \to N \to M$, where p is a conformal map with area growth (swelling) Ω_1 ($p: C \to N$ with Ω_1), q is a conformal map with area growth (shrinkage) Ω_2 ($q: N \to M$ with Ω_2), and N is a prescribed 3D shape at the swelled state (Fig. 1, Supplementary Fig. 1). The area growth functions have the relationships: $\Omega_1 > 1$, $\Omega_2 < 1$, and $\Omega = \Omega_1\Omega_2$. We prepare a 2D hydrogel C encoded with Ω using digital light lithography as described in Methods. The 2D hydrogel C transforms to the prescribed 3D shape at the swelled state N upon immersion in water ($T < T_c$) via $p: C \to N$ with Ω_1, where T_c is the volume phase transition temperature of pNIPAm hydrogels (~32.5 °C). Ω_1 is determined by Ω and the local degrees of swelling and shrinking of pNIPAm hydrogels: $\Omega_1 = (A_{25}/A_{35})\Omega$, where A_{25} and A_{35} are the local areas of hydrogels at 25 °C ($T < T_c$) and 35 °C ($T > T_c$), respectively. The 3D structure at the swelled

state N reversibly transforms to the target 3D shape M via $q: N \rightarrow M$ with Ω_2 upon temperature increase ($T > T_c$), for example, as shown in Fig. 1e and Supplementary Fig. 2, where $\Omega_2 = A_{35}/A_{25}$. The stresses developed by spatially-controlled in-plane growth Ω in 2D hydrogels are relieved by out-of-plane deformation, forming target 3D shapes, as bending is energetically more favorable than stretching in thin sheets[4-6]. We can rescale the size of the resulting 3D structure by uniformly rescaling the 2D printing area of Ω (e.g., Fig. 2k). However, as the size of a structure (or 2D printing area), or the ratio of the in-plane dimensions of a 2D hydrogel C to its thickness, increases, the sensitivity of shape morphing to the variations in shrinkage through the thickness increases, which can induce bending or rolling of the resulting 3D structure. For printing of large structures (e.g., face structure in Fig. 4e), we thus used fluorinated ethylene propylene film as a substrate and a cover to reduce the variations in the degrees of polymerization and crosslinking in the thickness direction of printed gels. For printing of structures larger than those in this work (e.g., 2D printing area larger than the size of our projection lithography cell with the dimension of 57 × 32 mm), the optimization of the thickness of hydrogels, or the ratio of the in-plane dimensions of C to its thickness, may be required.

Control of the degree of shrinkage of hydrogels

We control the degree of shrinkage of pNIPAm hydrogels by light exposure time using a precursor with two types of crosslinkers with different lengths: BIS (short-chain crosslinker) and PEGDA (long-chain crosslinker) as shown in Supplementary Fig. 4[4]. The dual crosslinking process forms a low density polymer framework with long-chain crosslinkers at an early stage of photopolymerization and photocrosslinking while continuously polymerizing monomers and crosslinking them with short-chain crosslinkers within the polymer framework throughout the

time course of polymerization and crosslinking reactions[4]. This process thereby increases the density of the polymer networks with light exposure time, which in turn decreases the degrees of swelling and shrinking of pNIPAm hydrogels (Supplementary Figs. 4 and 5)[4]. This approach differs from one that modulates the swelling of pNIPAm hydrogels by controlling their crosslink density with light irradiation dose[6].

Design of shape guiding modules

The shape-guiding modules with $K > 0$ used to form the stingray structures in Fig. 4 and Supplementary Figs. 17 and 19–21 have a growth function in the form

$$\Omega(r, \theta) = c\left[1 + a\left(\frac{r}{b(\theta)R}\right)^2\right]^{\alpha-1}$$

where r and θ are a radial and angular position, respectively, c, R, and α are constants, $a = \Omega_{\mathrm{r}}{}^{-1/(\alpha-1)} - 1$, and $b(\theta) = g/\sqrt{1-(1-g^2)\sin^2\theta}$. $\Omega_{\mathrm{r}} = \Omega_{\max}/\Omega_{\min}$, where $\Omega_{\max} = 0.76$ and $\Omega_{\min} = 0.36$. For the shape-guiding modules with low K in Fig. 4 and Supplementary Figs. 17 and 19–21, $c = 0.75\Omega_{\max}$, $\alpha = 0.8$, and $g = 0.27$ were used. For the shape-guiding module with high K in Supplementary Fig. 21, $c = \Omega_{\max}$, $\alpha = 0.8$, and $g = 0.27$ were used.

Two major modes of shape morphing of stingray structures

The stingray structures at two different phases of undulatory swimming motion shown in Fig. 4a (phase 0) and Supplementary Fig. 17 (phase 0.5) have two major modes of shape morphing (Fig. 4b, Supplementary Fig. 20). In the mode 1 without a shape-guiding module in both phase 0 (Supplementary Fig. 20a) and phase 0.5 (Supplementary Fig. 20b), the central body ($K > 0$) between the head ($K > 0$) and tail ($K > 0$) morphs downward ($k < 0$) in the direction opposite to

that of the head and tail ($k > 0$) along the major axis of the body (dotted green lines). As the pectoral fins ($K < 0$), or the principal curvatures of the fins along the major principal directions (indicated with solid green lines), should morph in the same direction as the central body ($k < 0$) along the interfaces of the body and the fins, the fins morph downward ($k < 0$) along the major principal directions (solid green lines), forming the configurations different from the target morphologies (mode 1). In the mode 2 with a shape-guiding module in both phase 0 (Supplementary Fig. 20a) and phase 0.5 (Supplementary Fig. 20b), the shape-guiding module with $K > 0$ in the central body directs the central body ($K > 0$) to morph upward ($k > 0$) in the same direction as the head and tail ($K > 0$), due to the inherent constraint (through-thickness variation in shrinkage). As the pectoral fins ($K < 0$), or the principal curvatures of the fins along the major principal directions (indicated with solid red lines in the fins), should morph in the same direction as the central body ($k > 0$) along the interfaces of the body and the fins, the fins morph upward ($k > 0$) along the major principal directions (solid red lines in the fins), forming the target morphologies (mode 2).

Supplementary References

1. Sawhney, R. & Crane, K. Boundary first flattening. *ACM Trans. Graph.* **37**, 5 (2017).
2. do Carmo, M. P. *Differential Geometry of Curves and Surfaces*. (Dover Publications, New York, 2016).
3. Springborn, B., Schrder, P. & Pinkall, U. Conformal equivalence of triangle meshes. *ACM Trans. Graph.* **27**, 77 (2008).
4. Nojoomi, A., Arslan, H., Lee, K. & Yum, K. Bioinspired 3D structures with programmable morphologies and motions. *Nat. Commun.* **9**, 3705 (2018).
5. Sharon, E. & Efrati, E. The mechanics of non-Euclidean plates. *Soft Matter* **6**, 5693-5704 (2010).
6. Kim, J., Hanna, J. A., Byun, M., Santangelo, C. D. & Hayward, R. C. Designing responsive buckled surfaces by halftone gel lithography. *Science* **335**, 1201-1205 (2012).
