# Supplementary material for: 2D material programming for 3D shaping
Source: Nat Commun. 2021 Jan 27;12:603. doi: 10.1038/s41467-021-20934-w (PMC7841157; doi:10.1038/s41467-021-20934-w)
Supplement: Supplementary file 3 — Description of Additional Supplementary Files [file 41467_2021_20934_MOESM3_ESM.pdf]

Description of Additional Supplementary Files

File Name: Supplementary Movie 1
Description: Formation of the automobile model shown in Fig. 1. This movie shows the shape transformation of a hydrogel sheet encoded with Ω for the automobile at a swelled state to the target shape at a shrunk state with time. The movie is shown 450x faster than real time.

File Name: Supplementary Movie 2
Description: Formation of a leaf structure. This movie shows the shape transformation of a hydrogel sheet encoded with Ω for the leaf at a swelled state to the target shape at a shrunk state with time. The movie is shown 450x faster than real time.

File Name: Supplementary Movie 3
Description: Reversible shape transformation of a hemisphere structure formed with a cone singularity. This movie shows the reversible shape transformation of the hemisphere between a shrunk state and a swelled state. The movie is shown 450x faster than real time.

File Name: Supplementary Movie 4
Description: Formation of a stingray structure at phase 0.5. This movie shows the shape transformation of a hydrogel sheet encoded with Ω with a shape-guiding module (low K) for the stingray at a swelled state to the target shape at a shrunk state. The movie is shown 450x faster than real time.

File Name: Supplementary Movie 5
Description: Reversible shape transformation of a stingray structure at phase 0. This movie shows multiple cycles of the shape transformation between the target shape at a shrunk state and the shape at a swelled state upon temperature change. The structure was formed using Ω with a shape-guiding module. The movie is shown 450x faster than real time.

File Name: Supplementary Movie 6
Description: Formation of the stingray model at phase 0.5 without and with shapeguiding modules. This movie shows the shape transformation of hydrogel sheets encoded with Ω (left) and Ω with a shape-guiding module (high K) (right) for the stingray at phase 0.5 at swelled states to the shapes at shrunk states with time. The movie is shown 450x faster than real time.

File Name: Supplementary Movie 7
Description: Formation of a face structure with a cone singularity. This movie shows the shape transformation of a hydrogel sheet encoded with Ω with a cone singularity for the face at a swelled state to the target shape at a shrunk state. The movie is shown 450x faster than real time.
